# Development of a Versatile Cancer Vaccine Format Targeting Antigen-Presenting Cells Using Proximity-Based Sortase A-Mediated Ligation of T-Cell Epitopes

**DOI:** 10.1021/acs.bioconjchem.4c00403

**Published:** 2024-11-07

**Authors:** Aru Z. Wang, Hendrik J. Brink, Rianne G. Bouma, Alsya J. Affandi, Maarten K. Nijen Twilhaar, Dijmphna A. M. Heijnen, Joelle van Elk, Janneke J. Maaskant, Veronique A. L. Konijn, Joeke G. C. Stolwijk, Hakan Kalay, Katarina Olesek, Yvette van Kooyk, Johan M. S. van der Schoot, Arthur E. H. Bentlage, Ferenc A. Scheeren, Martijn Verdoes, Gestur Vidarsson, Coenraad P. Kuijl, Joke M. M. den Haan

**Affiliations:** † Department of Molecular Cell Biology and Immunology, Amsterdam UMC Location Vrije Universiteit Amsterdam, De Boelelaan 1117, 1081 HV Amsterdam, The Netherlands; ‡ Cancer Center Amsterdam, Cancer Biology and Immunology, 1081 HV Amsterdam, The Netherlands; § Amsterdam Institute for Immunology and Infectious Diseases, 1081 HV Amsterdam, The Netherlands; ∥ Department of Medical Microbiology and Infection Control, Amsterdam UMC Location Vrije Universiteit Amsterdam, De Boelelaan 1117, 1081 HV Amsterdam, The Netherlands; ⊥ Department of Medical Biosciences, Institute for Chemical Immunology, Radboud University Medical Center, 6500 HB Nijmegen, The Netherlands; # Sanquin Research, 1066 CX Amsterdam, The Netherlands; ∇ Department of Biomolecular Mass Spectrometry and Proteomics, Utrecht Institute for Pharmaceutical Sciences and Bijvoet Center for Biomolecular Research, Utrecht University, 3508 TC Utrecht, The Netherlands; ○ Department of Dermatology, Leiden University Medical Center, 2333 ZA Leiden, The Netherlands

## Abstract

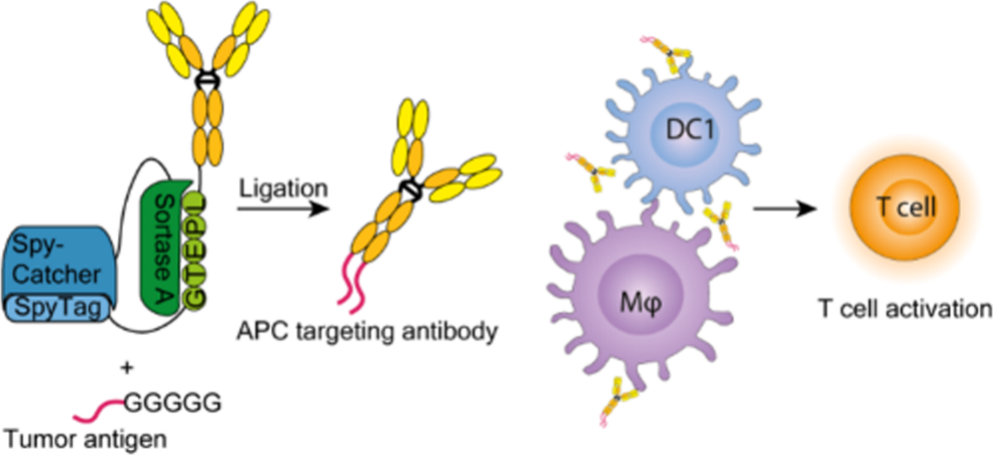

Cancer vaccines are a promising strategy to increase tumor-specific immune responses in patients who do not adequately respond to checkpoint inhibitors. Cancer vaccines that contain patient-specific tumor antigens are most effective but also necessitate the production of patient-specific vaccines. This study aims to develop a versatile cancer vaccine format in which patient-specific tumor antigens can be site-specifically conjugated by a proximity-based Sortase A (SrtA)-mediated ligation (PBSL) approach to antibodies that specifically bind to antigen-presenting cells to stimulate immune responses. DEC205 and CD169 are both receptors expressed on antigen-presenting cells that can be targeted to deliver antigens and stimulate T-cell responses. We used the CRISPR/HDR platform to produce mouse heavy chain IgG2a antibodies with DEC205 or CD169 specificity containing an SrtA recognition motif followed by a SpyTag at the C-terminus. Using a recombinant protein of SrtA linked to SpyCatcher, we applied proximity-based SrtA-mediated ligation to ligate fluorescein isothiocyanate (FITC)-labeled or antigenic peptides to the antibodies. Ligated antibodies bound to DEC205-expressing dendritic cells or CD169-expressing macrophages both *in vitro* and *in vivo*. More importantly, immunization with DEC205- or CD169-specific Abs linked to T-cell epitopes efficiently stimulated T-cell responses *in vivo*. To conclude, we have developed a cancer vaccine format using PBSL that enables the rapid incorporation of tumor antigens and could potentially be implemented for the synthesis of personalized cancer vaccines.

## Introduction

The treatment prospects of many cancer patients have been improved by cancer immunotherapies, such as checkpoint inhibitors that block the inhibitory receptors on T cells. However, a significant proportion of patients do not respond to these immunotherapies, which is correlated with an absence of immune cells in the tumor microenvironment, also described as cold tumors.^1−3^ Therapeutic cancer vaccines could be applied to improve the immune response of cold tumors and are expected to enhance the effects of checkpoint inhibitors.^4,5^

The primary elements of all cancer vaccines include tumor antigens produced by cancer cells and adjuvants that enhance dendritic cell maturation and T-cell activation. Cancer vaccines that contain patient-specific cancer antigens, so-called neo-antigens, have successfully been used in proof-of-concept studies for treating melanoma, pancreatic cancer and glioblastoma.^6−8^ While most recent clinical studies have utilized mRNA lipid nanoparticles for vaccination, clinical trials with synthetic long peptides have also been successful in achieving clinical responses.^8−13^ Moreover, a number of preclinical studies have investigated novel approaches to increase the immunogenicity of peptides by inclusion in nanodiscs or attachment to liposomes.^14,15^

The goal of cancer vaccines is to induce high frequencies of tumor-specific cytotoxic CD8^+^ T cells and CD4^+^ T cells, which is mostly achieved after uptake, processing, and presentation of tumor antigens by dendritic cell (DCs). For most vaccine platforms, uptake by DCs is mediated by nonspecific mechanisms. Remarkably, antigen-presenting cell (APC)-targeted antibody (Abs)-antigen conjugates specific for uptake receptors on cDC1, such as DEC205 and DNGR-1/Clec9A, have been shown to enhance the uptake and T-cell priming capacity significantly.^16−20^ This strategy not only improves the Ag presentation but also tailors the immune response, making it a valuable tool in vaccine development and immunotherapy. In our previous studies, we have investigated Ab-mediated antigen targeting to splenic CD169^+^ macrophages and discovered that these cells efficiently transfer antigens to cross-presenting DCs and thereby stimulate CD8^+^ T-cell responses and antitumor reactivity.^21−23^

Both chemical and enzymatic methods can by employed to conjugate antigenic peptides or proteins to Abs.^24−26^ Conventional chemical conjugation methods, such as *N*-hydroxysuccinimide (NHS) ester chemistry, suffer from limitations in conjugation site specificity and stoichiometry control, leading to heterogeneous Abs conjugates with potentially compromised functionality.^27^ In contrast, Sortase A (SrtA)-mediated Ab conjugation enables site-specific and controlled attachment of cargo molecules to Abs that contain the SrtA recognition motif, resulting in homogeneous conjugates with preserved Ab binding affinity and biological activity.^20,28−33^ However, a major limitation of SrtA is its low binding affinity to the recognition motif, poor reaction kinetics, and the need for high concentrations of the cargo molecule. Wang et al., introduced the proximity-based Sortase A mediated ligation (PBSL) technique as a solution to address these limitations.^34,35^ PBSL incorporates the SpyTag-SpyCatcher protein pair system to enhance the interaction of SrtA with the recognition motif. Once the SpyTag-SpyCatcher protein pair comes into contact it will rapidly form an irreversible isopeptide bond.^36^ For PBSL, a SpyTag is introduced near the SrtA recognition domain and the SrtA is linked to the SpyCatcher protein to enable high affinity interaction which significantly increases the ligation efficiency.^31^ Our study demonstrates that PBSL can be applied to site-specifically conjugate T-cell epitopes to DC- and macrophage-specific Abs. Conjugated Abs were specifically taken up by these cells and induced antigen-specific CD8^+^ and CD4^+^ effector T-cell immune responses.

## Results and Discussion

### Engineering of CD169-, DEC205-Specific, and Isotype Mouse IgG2a_silent_ Fc with LPETG and SpyTag Hybridomas

**Figure 1:**
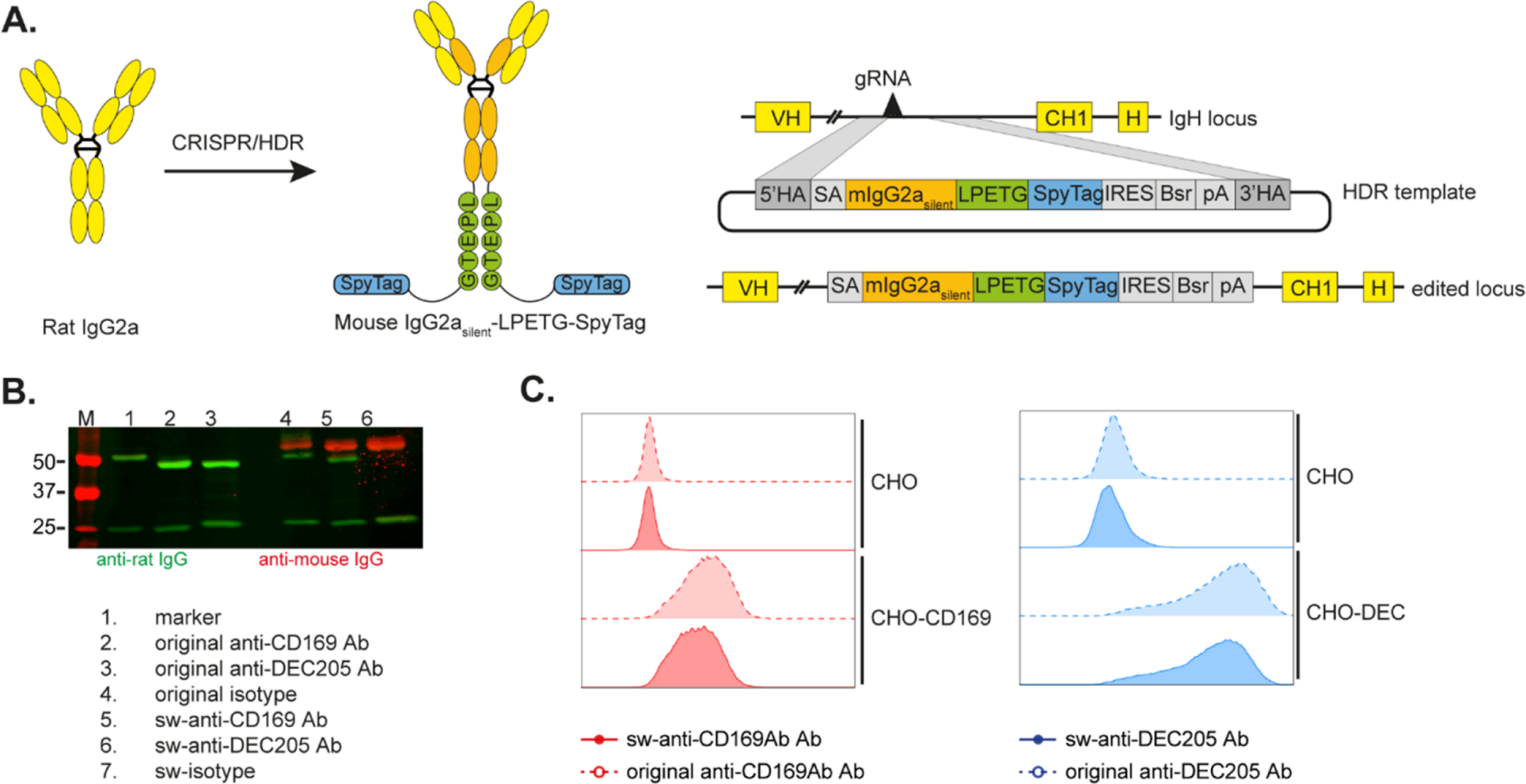
CRISPR/HDR engineering of CD169-, DEC205-specific, and isotype control rat IgG2a hybridomas to produce mouse IgG2a with SrtA recognition motif and SpyTag. (A) Hybridomas producing rat IgG2a Abs specific for mouse CD169 or DEC205 were engineered with CRISPR/HDR to produce murine mIgG2a_silent_ isotype Abs containing the SrtA recognition site LPETG and SpyTag. (B) Western blot of the original rat IgG2a and the switched (sw)-mouse IgG2a Abs specific for mouse CD169, DEC205, and isotype control. The blot was incubated with anti-rat (green) and anti-mouse secondary Ab (red). (C) Flow cytometry analysis of isotype-sw-Abs bound to CHO cells that express CD169 or DEC205. Anti-rat IgG Abs and anti-mouse IgG Abs were used to detect the original Abs or the sw-Abs, respectively.

Rat IgG2a hybridomas specific for CD169, DEC205, and an isotype control, were genetically modified to generate recombinant hybridomas capable of secreting the murine mIgG2a silent (L234A/L235A/N297A) isotype which has reduced affinity for murine FcγRs, utilizing the CRISPR/HDR technique outlined by van der Schoot et al.^37^ This resulted in the in-frame insertion of a splice acceptor (SA), the mIgG2a Fc region (depicted in orange), an LPETG motif (depicted in green), a SpyTag motif (depicted in blue), an IRES element, a blasticidin-resistance gene (Bsr), and a polyA transcription termination signal (pA) upstream of the native CH1 (Figure 1A).

Following the selection of clones, the CRISPR/HDR-modified sw-Abs were analyzed by Western blot using anti-rat and anti-mouse secondary Abs and compared to the original Abs. The original Abs (lanes 1, 2, and 3) were of rat origin, as evidenced by the green bands for ∼50 kDa heavy chain (HC) and ∼25 kDa light chain (LC) detected by goat anti-rat secondary Abs. In contrast, the sw-Abs (lanes 4–6) contained a murine IgG HC (red bands), while the light chain remained of rat origin (green bands) (Figure 1B). However, we could still observe small amounts of rat HC green bands in lanes 4 and 6 in multiple clones tested (data not shown). This suggests that the rat HC downstream of the inserted murine HC could still be transcribed due to an incomplete transcription termination as previously described.^37^ However, since these rat HC do not contain the SpyTag, these HC will not be involved in the PBSL reaction and therefore will not be part of the end products.

The binding capacity of the original Abs and sw-Abs was assessed by flow cytometry with CHO cells expressing CD169 or DEC205. The original Abs and sw-Abs exhibited similar binding profiles (Figure 1C), which indicates that the CRISPR/HDR engineering process did not alter the binding ability of the Abs.

### Optimization of PBSL Conditions for Ab Conjugation

**Figure 2:**
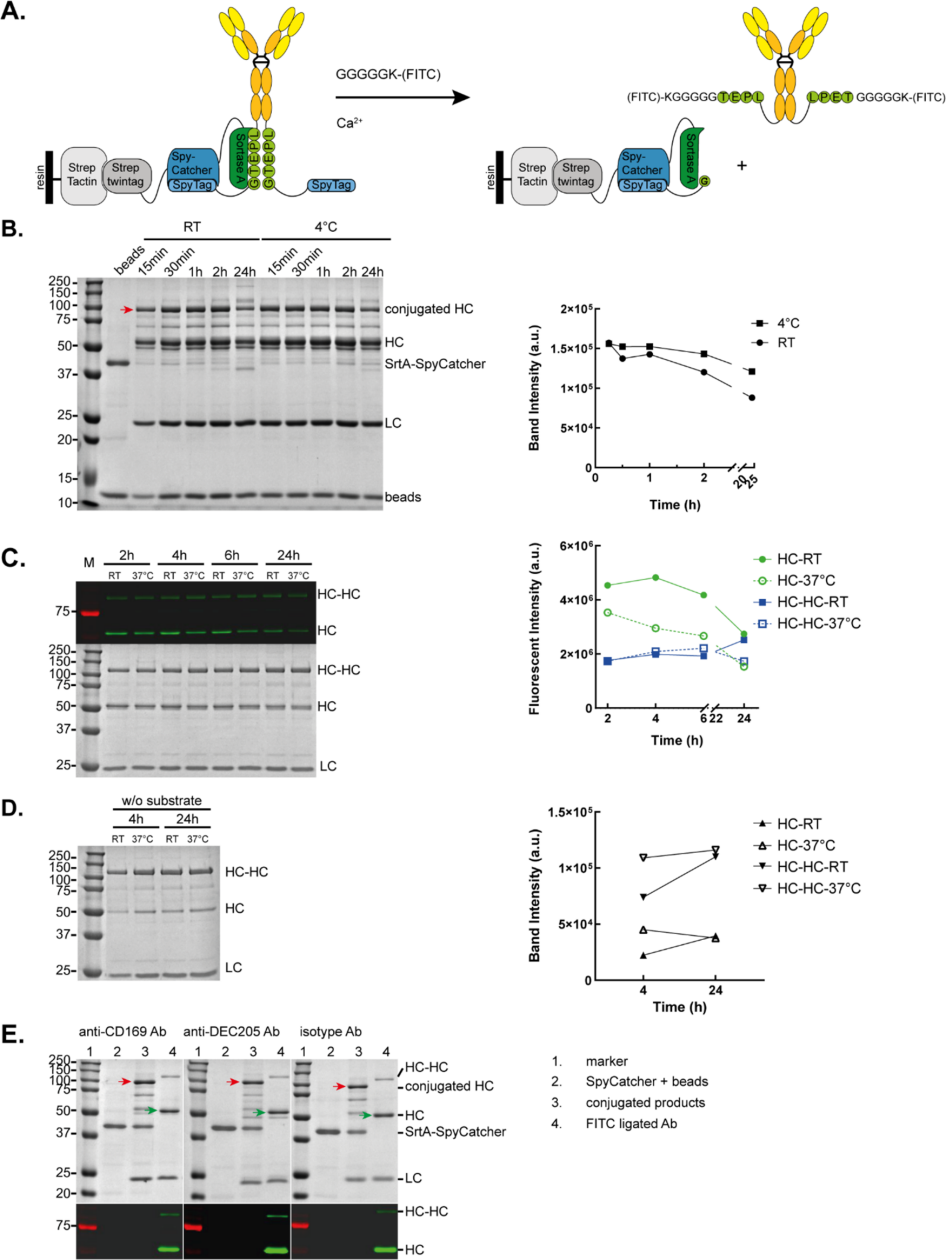
Optimization of PBSL reaction of fluorescent substrate to sw-mouse IgG2a Abs. (A) Schematic diagram of ligation of fluorescent GGGGGK-(FITC) probe to Ab-HC C-terminus by PBSL. (B) Investigation of different incubation temperatures and times for conjugation of Abs to SrtA-SpyCatcher protein using InstantBlue-stained SDS-PAGE gel (left panel). The relative intensity values of the conjugated HC were quantified and normalized to the bead band intensity (right panel). (C) Analysis of different incubation temperatures and times for the SrtA ligation using the GGGGGK-(FITC) substrate using fluorescent imaging or InstantBlue-stained SDS-PAGE gel (left panel). Reactions were assessed at RT and 37 °C and after 2, 4, 6, and 24 h and quantified (right panel). (D) SDS-PAGE gel of SrtA-SpyCatcher reaction with Ab-LPETG-SpyTag in the absence of a FITC peptide substrate. The relative intensity values of the HC were quantified and normalized to the LC intensity value (right panel). (E) Representative SDS-PAGE visualizing PBSL labeling of anti-CD169-LPETG-SpyTag, anti-DEC205-LPETG-SpyTag, and isotype-LPETG-SpyTag with GGGGGK-(FITC).

To determine the optimal conditions for PBSL of Abs, a FITC-labeled peptide was employed as a model substrate (Figure 2A). First, the SrtA-SpyCatcher fused with a streptavidin tag was purified by affinity chromatography using Strep-Tactin resin beads (Figure 2B-first lane).

Using equimolar amounts of each product, the efficiency of conjugation of sw-anti-CD169 Abs to SrtA-SpyCatcher beads was evaluated at room temperature (RT) and 4 °C. At multiple time points (15, 30 min, 1, 2, and 24 h), samples were collected for analysis. The conjugated HC band of ∼80 kDa (red arrow), corresponding to the HC-LPETG-SpyTag (∼50 kDa) plus the SrtA-SpyCatcher protein (∼29.9 kDa), was observed on the gel. The relative band intensity of the conjugated HC, normalized to the beads’ intensity, revealed that conjugation occurred within 15 min. The conjugation efficiency at 4 °C and RT was comparable at 15 min; however, conjugation at 4 °C maintained higher conjugated HC levels at later time points (Figure 2B). Based on these results, we decided to use a 1 h incubation step at 4 °C for the conjugation of the SpyTag-SpyCatcher protein pair.

To optimize the ligation conditions of the SrtA reaction, we investigated different incubation temperatures (RT and 37 °C). Equal volumes of ligated products were collected at various time points (2–24 h). The relative fluorescence signal was quantified and normalized to the LC intensity after InstantBlue staining (Figure 2C). Analysis of the fluorescence revealed that FITC-ligation of HC was most efficiently achieved after a 2 h SrtA reaction at RT compared to reactions performed at 37 °C and that the ligation efficiency decreased over time. Next to the desired FITC-ligated HC, a band of two HC was visible, which was likely caused by a SrtA-mediated ligation between the two HC chains as has been described.^38^ Analysis of the fluorescent intensity of the HC and cross-linked HC-HC bands revealed that the presence of unwanted cross-linked HC-HC-FITC bands increased with prolonged incubation times, indicating that 2 h at RT was optimal for obtaining the desired ligated Abs while minimizing the formation of side products (Figure 2C).

In addition, SrtA reactions without the FITC substrate were conducted to detect SrtA-mediated hydrolysis and cross-linking of the HC. The relative band intensities of the hydrolyzed HC and cross-linked HC-HC products, normalized to the LC intensity, were quantified and plotted (Figure 2D). After 4 h at RT, the formation of both the hydrolyzed HC and cross-linked HC-HC products was lower compared with the reactions performed at 37 °C and for 24 h. To minimize undesired hydrolysis and cross-linking HC reactions, we chose to use 2 h at RT as the optimal conditions for ligation in the further experiments.

Next, anti-CD169, anti-DEC205, and isotype control Abs were ligated with FITC-labeled peptide using the optimized conditions (Figure 2E). The bound SpyCatcher and bead resin were observed in Figure 2E-lane 2. Following the conjugation step, an ∼80 kDa molecular-weight band (red arrow) corresponding to the conjugated Ab-LPETG-SpyTag to SrtA-SpyCatcher protein was detected (Figure 2E-Lane 3). After the SrtA ligation reaction, a fluorescent HC band (green arrow) corresponding to the desired ligated product was detected (Figure 2E-Lane 4). Since the ligation of FITC resulted in a decrease in size (Table S1), we compared the FITC-ligated Ab with the sw-Ab on gel. After ligation, sw-Ab was not anymore detected, and the product consisted of single HC ligated to FITC and a minor portion of cross-linked HC-HC ligated to FITC (Figure S1A), representing approximately 70% of sw-Ab ligated to 2 peptides and 25–30% of cross-linked sw-Ab ligated to one FITC molecule. To check whether there was still nonligated SpyTag-containing HC present, the ligated product was incubated with SpyCatcher-mCherry overnight and analyzed by SDS-PAGE under non- and denaturing conditions (Figure S1B). While the control sw-Ab could bind to the SpyCatcher, which resulted in a complex of approximately 236 kDa, ligated sw-Ab did not bind SpyCatcher (nondenatured gel). This indicates that all HC have reacted and that approximately 70% of the Abs contain two FITC molecules and 25–30% contain one FITC molecule. In summary, we have established the conditions for the PBSL procedure and confirmed its effectiveness by using FITC molecules.

### *In Vitro* and *In Vivo* Binding and Uptake of FITC-Ligated Abs

**Figure 3:**
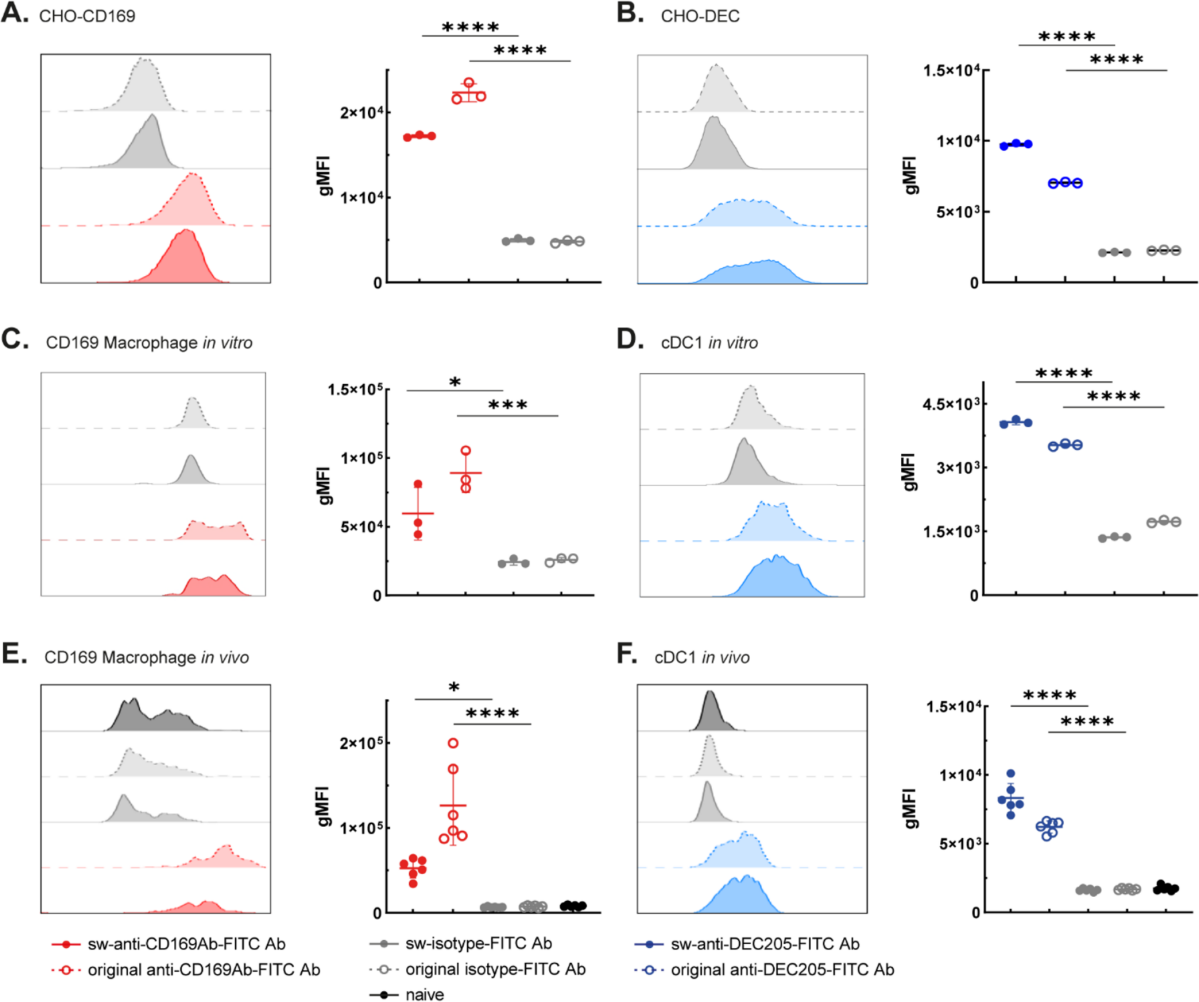
PBSL of FITC to sw-Abs result in functional Abs that bind to their ligands *in vitro* and *in vivo.* (A, B) Binding of PBSL-mediated FITC-ligated or NHS-labeled anti-CD169, anti-DEC205 Abs, and isotype controls to CD169 (A) or DEC205-expressing (B) CHO cells was determined by flow cytometry. (C, D) *In vitro* binding of PBSL-mediated FITC-ligated Abs and NHS-labeled original Abs to splenic CD169 macrophages (C) and cDC1 (D) were determined by flow cytometry. (E, F) *In vivo* binding of PBSL-mediated FITC-ligated Abs and NHS-labeled original Abs to splenic CD169 macrophages (E) and cDC1 (F) were determined 30 min after intravenous (i.v.) injection by flow cytometry. Mice were injected (i.v.) with 20 μg sw-anti-CD169-FITC, sw-anti-DEC205-FITC, sw-isotype-FITC or NHS-labeled original anti-CD169-FITC, original anti-DEC205-FITC, original isotype-FITC. Indicated are representative histogram overlays from triplicates (A–D) or 6 mice from two independent experiments (E, F) and geometric mean fluorescence intensity (gMFI) quantified and indicated as mean ± SD. Statistical analysis one-way ANOVA with Šidák’s multiple comparison test with respective isotype controls. *p < 0.05, ***p < 0.001, ****p < 0.0001.

The binding and uptake of FITC-ligated Abs was evaluated *in vitro* and after injection *in vivo* by flow cytometry. Sw-anti-CD169-FITC and sw-anti-DEC205-FITC Abs bound to CHO cells expressing CD169 or DEC205, respectively, and the binding was comparable to the directly NHS-labeled original Abs (Figure 3A,B). Next, ligated Abs were incubated with splenocytes that contain CD169^+^ macrophages (MQ) and DEC205^+^ cDC1 (gating strategy in Figure S2). Both NHS-labeled and ligated anti-CD169-FITC and anti-DEC205-FITC Abs showed significantly enhanced binding compared to the isotype control (Figure 3C,D). For *in vivo* Ab uptake, mice were intravenously injected with 20 μg of ligated anti-CD169-FITC, anti-DEC205-FITC, isotype control, or NHS-labeled original Abs, and the fluorescent signal in multiple splenic immune cell subsets was evaluated 30 min after injection. Specific binding of sw-anti-CD169-FITC and sw-anti-DEC205-FITC to splenic CD169^+^ MQ and DEC205^+^ cDC1 cells was detected, respectively, when compared to the corresponding isotype controls (Figure 3E,F). These results indicate that PBSL can be used to fluorescently label Abs without affecting their binding function.

### T-Cell Epitopes Ligation to sw-Abs Induce Antigen-Specific T-Cell Priming

**Figure 4:**
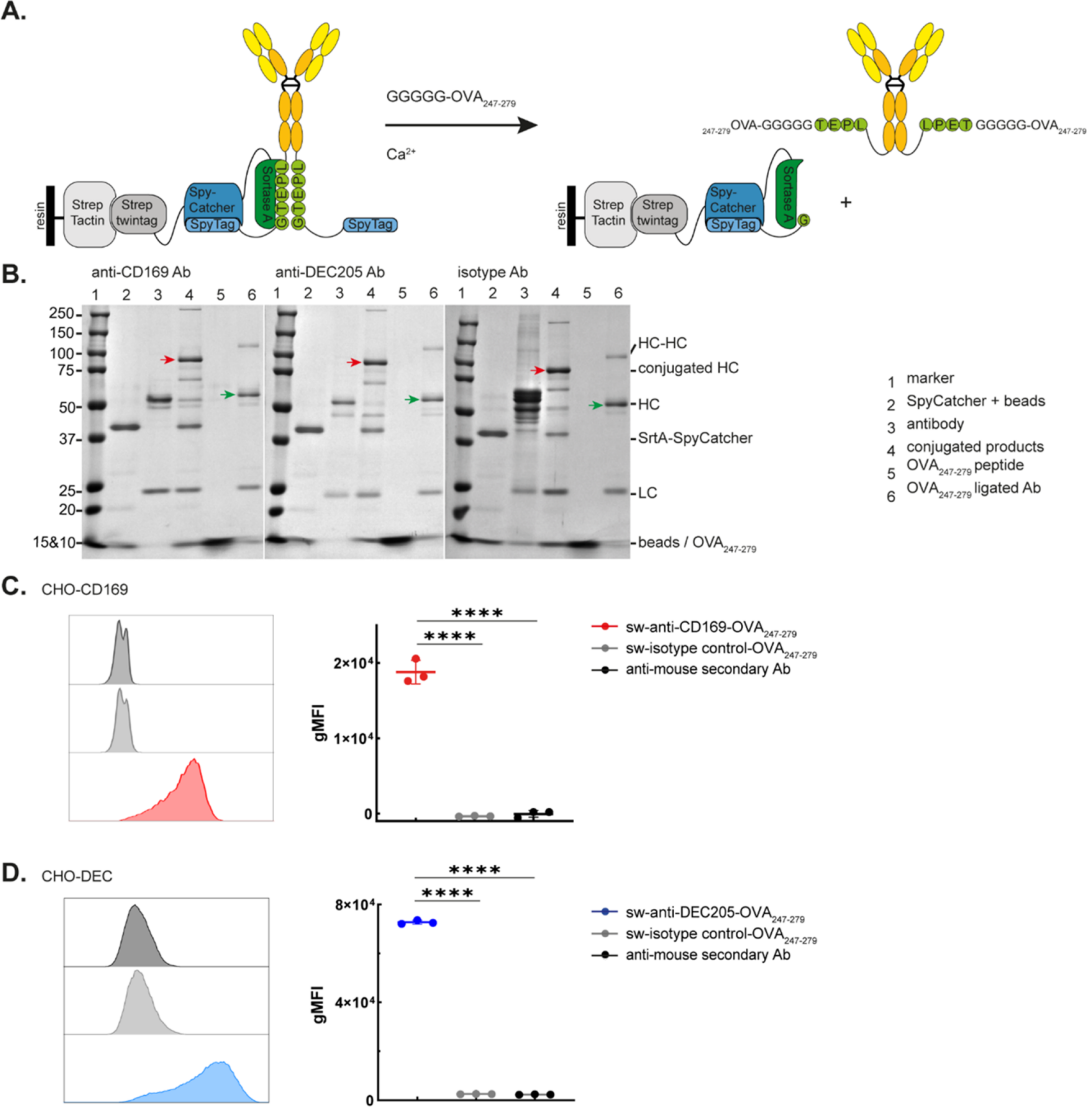
PBSL of OVA_247–279_ peptides to sw-anti-CD169, anti-DEC205, and isotype control mouse IgG2a Abs. (A) Schematic diagram of GGGGG-OVA_247–279_ peptide ligation to sw-mouse IgG2a containing SrtA and SpyTag motifs by PBSL. (B) Representative SDS-PAGE visualizing the different steps of the PBSL procedure of GGGGGOVA_247–279_ peptide to the sw-anti-CD169, anti-DEC205, and isotype mouse IgG2a Abs. The cross-linked HC-HC, conjugated HC (red arrow), original HC, peptide-ligated HC (green arrow), SrtA-SpyCatcher, and LC were labeled adjacent to the corresponding protein bands on the gel image. (C, D) CHO cells expressing CD169 or DEC205 were stained with PBSL-ligated anti-CD169-OVA_247–279_, anti-DEC205-OVA_247–279_, and isotype-OVA_247–279_ Abs. The gMFI signals of the anti-mouse secondary Ab were determined by flow cytometry. Representative histogram and graphs with mean ± SD from triplicates were shown. Statistical analysis one-way ANOVA with Šidák’s multiple comparison test. ****p < 0.0001.

Next, model antigen ovalbumin (OVA_247–279_) was ligated to the sw-Abs. OVA_247–279_ peptide containing a CD8^+^ T-cell epitope and a CD4^+^ T-cell epitope was ligated to sw-CD169, sw-DEC205, and isotype control Abs in order to induce antigen-specific T-cell priming. The schematic diagram illustrates the ligation of the GGGGG-OVA_247–279_ (4.2 kDa) to the sw-Abs (Figure 4A). SDS-PAGE analysis confirmed the successful PBSL of sw-CD169-, DEC205-, and isotype control Abs with OVA peptides by the 1.6 kDa molecular weight increase (Figure 4B: lane 3 vs lane 6 and Table S1). Similar to the FITC-ligated Abs, about 70% of sw-Ab was ligated to two OVA_247–279_ peptides, while 25–30% was ligated to one. Band intensity ratios from InstantBlue-stained gels were used to determine these percentages. (Figure S1A). We evaluated the binding of OVA_247–279_-ligated Abs to their respective ligands using an anti-mouse secondary Ab. The OVA_247–279_-ligated Abs showed significant binding to CHO cells expressing CD169 or DEC205. (Figure 4C,D).

**Figure 5:**
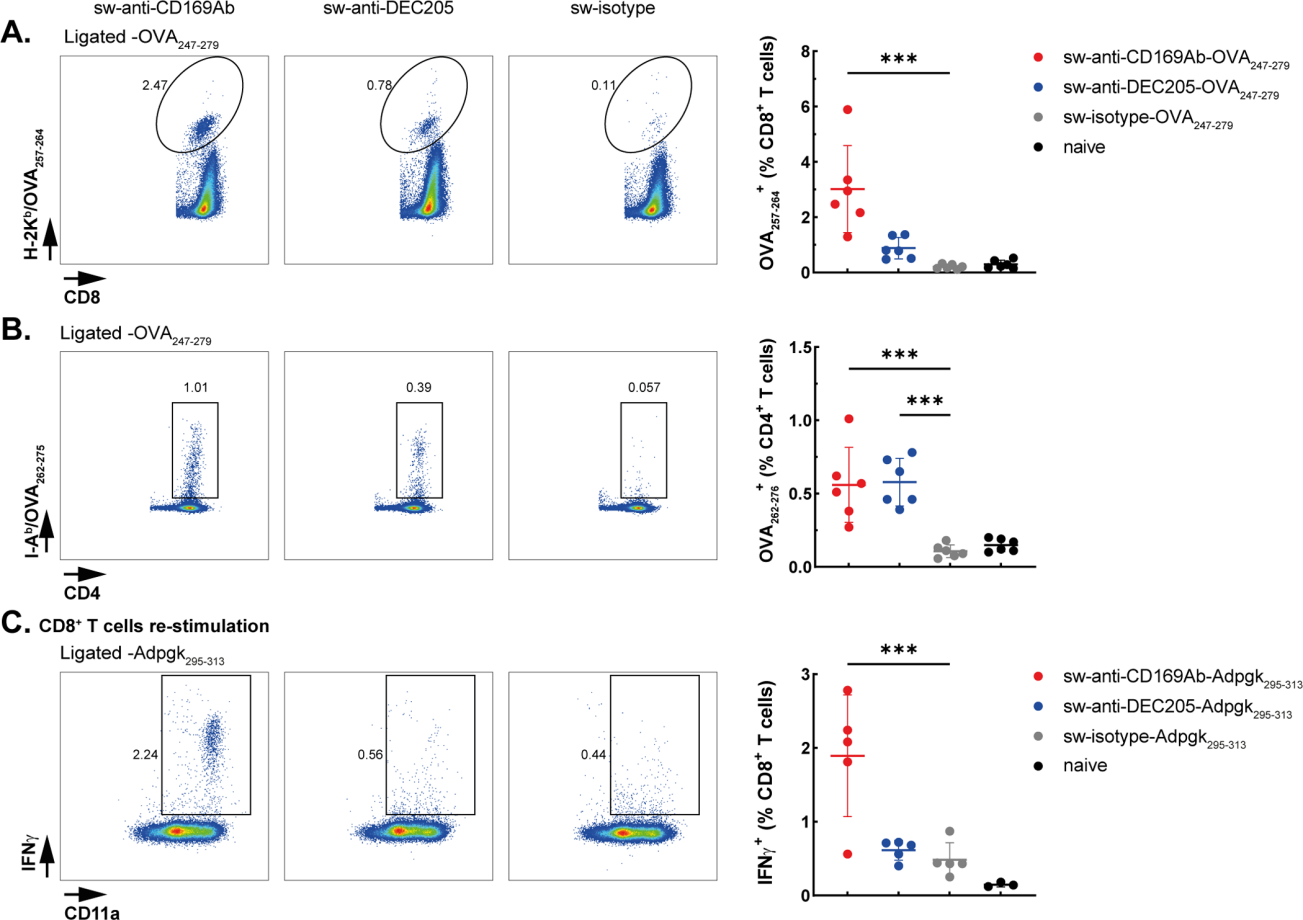
Immunization with anti-CD169-OVA_247–279_ and anti-CD169-Adpgk_295-313_ Abs induces robust CD8^+^ and CD4^+^ T-cell responses. Mice were immunized with 3 μg of ligated antibodies, 25 μg of anti-CD40 antibody, and 25 μg of Poly(I:C), with immune responses assessed on day 7. (A, B) Splenic OVA-specific CD8^+^ and CD4^+^ T-cell responses were evaluated using H-2K^b^-OVA_257–264_ tetramer and I-A^b^-OVA_262–276_ tetramer staining, respectively, with representative dot plots shown alongside quantification of frequencies (mean ± SD; *n* = 6, pooled from two experiments). (C) Adpgk_295–313_-specific CD8^+^ T-cell activation was measured after 5 h of restimulation with Adpgk_299–307_ peptide, and percentage of IFNγ-producing cells is displayed (mean ± SD; *n* = 5). Statistical analysis one-way ANOVA with Šidák’s multiple comparison test. ***p < 0.001.

To evaluate the induction of immune responses *in vivo*, mice were immunized with 3 μg of OVA_247–279_ ligated sw-anti-CD169, anti-DEC205, or isotype control in the presence of adjuvant (25 μg of anti-CD40 Ab and 25 μg of Poly(I:C)). Seven days after immunization OVA-specific CD8^+^ and CD4^+^ T-cell responses were evaluated using H-2K^b^-OVA_257–264_ and I-A^b^-OVA_262–276_ tetramer (Figure 5A and gating strategy in Figure S3A,B) and intracellular IFNγ staining, respectively (Figure S4 and gating strategy in Figure S3C). A strong induction of antigen-specific CD8^+^ T cells and IFNγ secretion were observed after targeting OVA_247–279_ peptides to CD169^+^ macrophages, while the CD8^+^ T-cell response and IFNγ secretion after targeting to DEC205 was low (Figures 5A and S4). Interestingly, both sw-anti-CD169-OVA and anti-DEC205-OVA induced OVA-specific CD4^+^ T-cell responses (Figure 5B) as detected by tetramer staining.

Finally, Adpgk_295–313_ containing a neoantigen expressed in the colorectal cancer cell line MC-38,^39,40^ was ligated to the sw-Abs, and the binding functions of sw-Abs were verified using CHO cells expressing CD169 or DEC205 (Figure S5). Approximately 70% of sw-Ab ligated to two Adpgk_295–313_ peptides and 25–30% to one. These ratios were derived from band intensities on InstantBlue-stained gels (Figure S1A). Mice were immunized with 3 μg of Adpgk_295–313_ ligated antibodies in the presence of adjuvant, and antigen-specific CD8^+^ T-cell responses were detected by intracellular IFNγ staining 7 days after the immunization (Figure 5C). Again, strong T-cell responses were detected after targeting to CD169^+^ APCs, demonstrating the feasibility of PBSL to generate Ab-antigen conjugates for vaccination purposes. DEC205 targeting of Adpgk_295–313_ resulted in a low CD8^+^ T-cell response, which was lower than expected. Since we used a murine mIgG2a containing mutations (L234A/L235A/N297A) to decrease FcγR binding and a recent study indicated that FcRn binding was important for DEC205-mediated CD8^+^ T-cell cross-priming,^37,41^ we evaluated FcRn binding of the mutated mIgG2a. However, the mutations in mIgG2a did not affect FcRn binding (Figure S6) and the underlying mechanism of the reduced CD8^+^ T-cell response to ligated anti-DEC205 remains insufficiently understood and requires further investigation. However, the experiments with CD169-targeting Ab indicate that PBSL ligation to Abs is feasible and can be used to produce cancer vaccines that stimulate antigen-specific T-cell responses.

## Conclusions

In this study, we have developed a versatile cancer vaccine platform that allows efficient conjugation of tumor antigens via PBSL to Abs to deliver these antigens to APCs. To achieve this, we employed a CRISPR/HDR–based strategy to generate recombinant hybridomas secreting sw-mouse IgG2a Fc silent Abs with a LPETG and SpyTag at the C-terminus. First, we ligated an FITC-containing peptide to the anti-CD169 and anti-DEC205 Abs and demonstrated that these FITC-ligated Abs retained their binding functionality both *in vitro* and *in vivo*. Subsequently, we ligated an OVA-derived peptide and a neoantigen Adpgk_295–313_ containing a long peptide to the anti-CD169 or anti-DEC205 Abs and observed T-cell priming *in vivo*. Our results demonstrate that PBSL enables site-specific conjugation of T-cell epitopes to APC-specific Abs, which were specifically taken up *in vivo* and induced antigen-specific CD8^+^ and CD4^+^ effector T-cell immune responses. The versatility of this platform allows for the conjugation of neo-antigens in the form of peptides, making it adaptable to personalized therapy. In conclusion, our study provides a potential approach for developing personalized cancer vaccines by utilizing the targeting properties of APC-specific antibodies alongside PBSL for precise antigen conjugation.

## Supporting Information Available

Experimental methods and materials; nucleotide sequences; amino acid sequences; gating strategy employed to identify CD169 macrophages and cDC1; gating strategy employed to identify T cells; IFNγ data; PBSL of Adpgk_295-313_ peptides to sw-Abs; Abs binding affinity to mouse FcRn; and anti-mouse antibodies for staining (PDF)
