# Supplementary material for: Development of a Versatile Cancer Vaccine Format Targeting Antigen-Presenting Cells Using Proximity-Based Sortase A-Mediated Ligation of T-Cell Epitopes
Source: Bioconjug Chem. 2024 Nov 7;35(11):1805–14. doi: 10.1021/acs.bioconjchem.4c00403 (PMC11583207; doi:10.1021/acs.bioconjchem.4c00403)
Supplement: Supplementary file 1 — bc4c00403_si_001.pdf [file bc4c00403_si_001.pdf]

Supporting Information

Development of a versatile cancer vaccine format targeting antigen presenting cells using proximity-based Sortase A mediated ligation of T cell epitopes

Aru Z. Wang,[†, ‡, §] Hendrik J. Brink,[†, ‡, §, #] Rianne G. Bouma,[†, ‡, §, #] Alsya J. Affandi,[†, ‡, §] Maarten K. Nijen Twilhaar,[†, ‡, §] Dijmphna A.M. Heijnen,[†, ‡, §] Joelle van Elk,[†, ‡, §] Janneke J. Maaskant,[&] Veronique A.L. Konijn,[†, ‡, §] Joeke G.C. Stolwijk,[†, ‡, §] Hakan Kalay,[†, ‡, §] Katarina Olesek,[†, ‡, §] Yvette van Kooyk,[†, ‡, §] Johan M.S. van der Schoot,[¶] Arthur E.H Bentlage,[‖] Ferenc A. Scheeren,[^] Martijn Verdoes,[¶] Gestur Vidarsson,[‖, ⊥] Coenraad P. Kuijl,[&] and Joke M.M. den Haan,[†, ‡, §, *]

† Amsterdam UMC location Vrije Universiteit Amsterdam, Department of Molecular Cell Biology and Immunology, De Boelelaan 1117, 1081 HV, Amsterdam, The Netherlands

‡ Cancer Center Amsterdam, Cancer biology and Immunology, 1081 HV, Amsterdam, The Netherlands

§ Amsterdam institute for Immunology and Infectious Diseases, 1081 HV, Amsterdam, The Netherlands

& Amsterdam UMC location Vrije Universiteit Amsterdam, Department of Medical Microbiology and Infection Control, De Boelelaan 1117, 1081 HV, Amsterdam, The Netherlands

¶ Radboud University Medical Center, Department of Medical Biosciences, Institute for Chemical Immunology, 6500 HB, Nijmegen, The Netherlands

‖ Sanquin Research, Amsterdam, 1066 CX, The Netherlands

⊥ Department of Biomolecular Mass Spectrometry and Proteomics, Utrecht Institute for Pharmaceutical Sciences and Bijvoet Center for Biomolecular Research, Utrecht University, 3508 TC, Utrecht, The Netherlands

^ Department of Dermatology, Leiden University Medical Center, 2333 ZA, Leiden, The Netherlands

# shared second author

* Correspondence should be sent to j.denhaan@amsterdamumc.nl

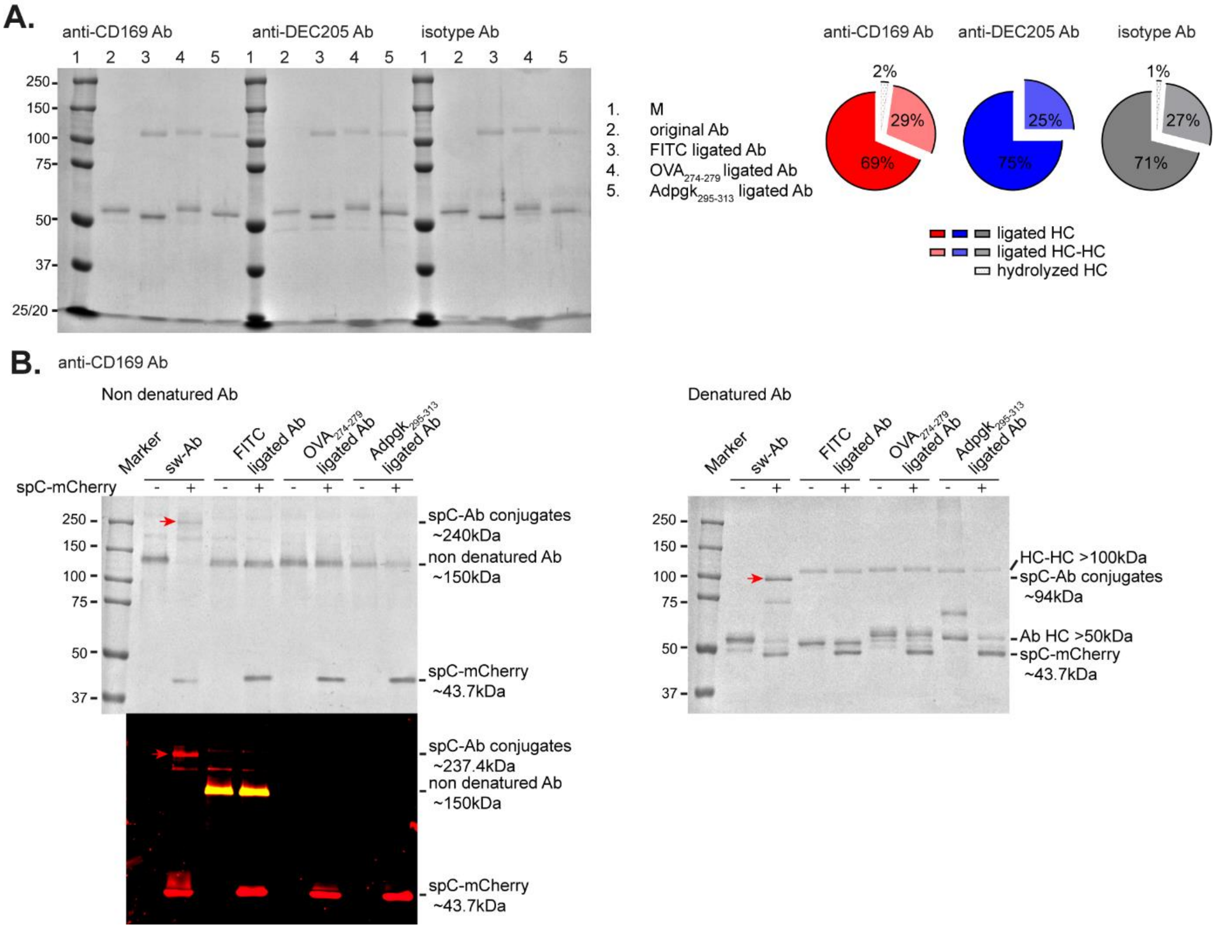

Figure S1. A. Overview of sw-Abs and ligated sw-Abs on an SDS-PAGE gel. Pie chart showing the mean percentage of ligated Abs categorized in HC ligated with peptides, cross-linked HC ligated with peptide and hydrolyzed HC without peptide. Relative ratios were calculated by dividing the intensity of each component band by the total intensity of ligated HC, HC-HC, and hydrolyzed bands in each sample, with percentages representing the average from three ligated Abs, indicating ligation efficiency. B. Detection of SpyCatcher conjugation to SpyTag on sw-Abs, ligated Abs, with anti-CD169 Ab as a representative example. 1 µM of each spC-mCherry and Abs were incubated overnight at 4°C and 0.5ug Abs were loaded on gel.

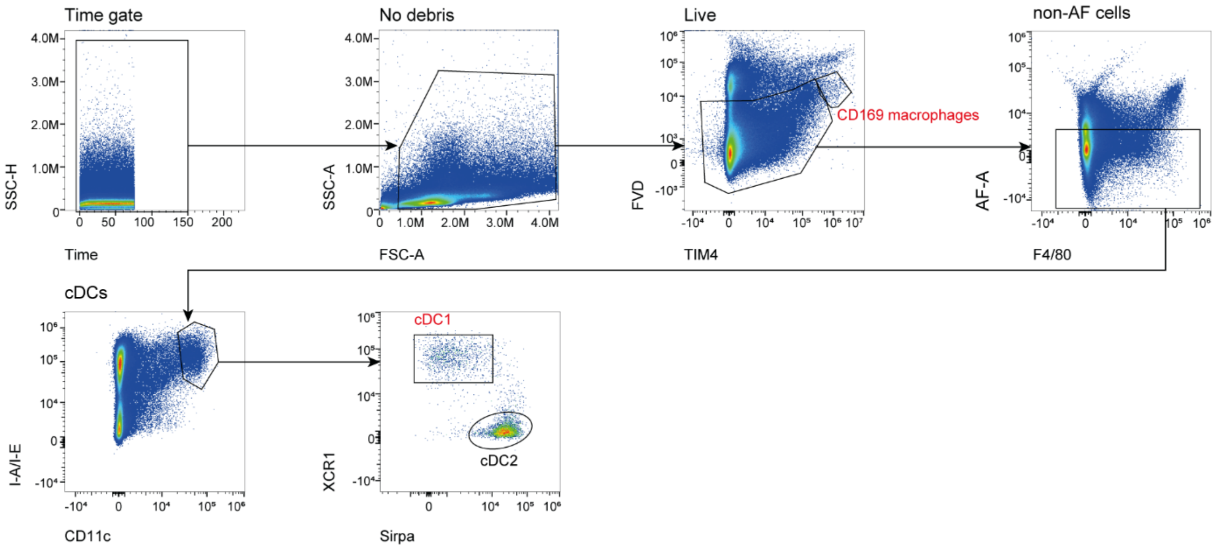

Figure S2. Gating strategy employed to identify CD169 macrophages and cDC1, related to Figure 3.

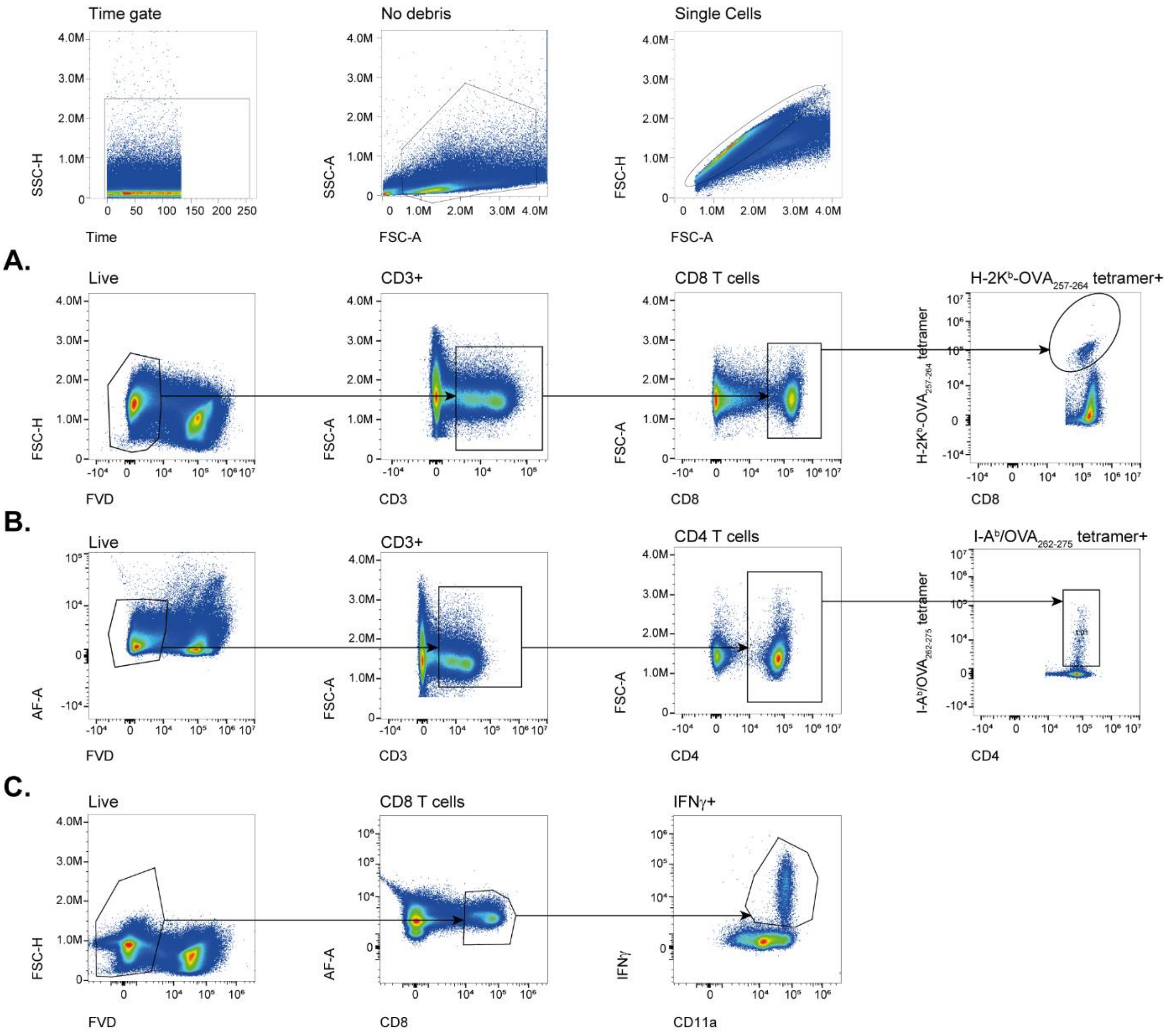

Figure S3. Gating strategy of isolated splenocytes used to evaluate adaptive immune responses, related to Figure 5 and Figure S3. Top panel shows initial gating to obtain single cell fraction. A. Gating strategy for H-2K^{b}-OVA$_{257-264}$ tetramer^{+} CD8^{+} T cells. B. Gating strategy for I-A^{b}-OVA$_{262-276}$ tetramer^{+} CD4^{+} T cells. C. Gating strategy for IFNγ-producing CD8^{+} T cells, related to Figure 5C and Figure S4.

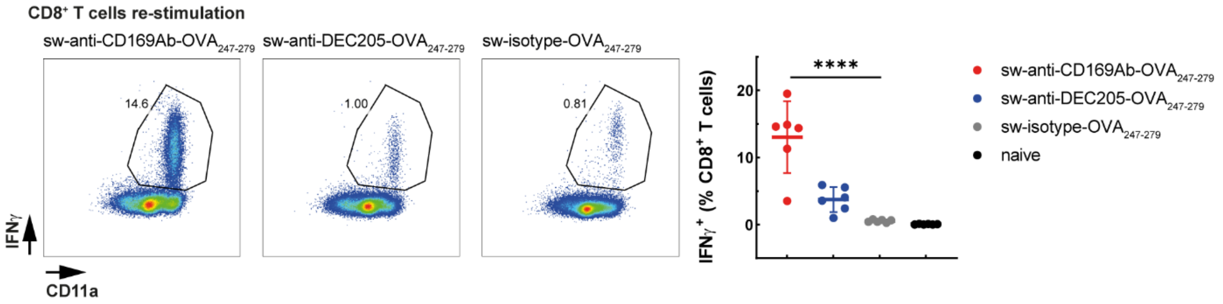

Figure S4. Splenocytes from mice which were immunized with 3 µg of $OVA_{247-279}$ ligated sw-anti-CD169, anti-DEC205, or isotype control in the presence of adjuvant (25 µg anti-CD40 Ab and 25 µg Poly(I:C)) were restimulated with $OVA_{257-264}$ peptide for 5 h, and IFNγ-producing $CD8^+$ T cells were detected with intracellular flow cytometry staining (related to Figure 5A). Indicated is the percentage of IFNγ-expressing CD8 T cells ± SD (n = 6) (pooled from 2 independent experiments). Statistical analysis one-way ANOVA with Šidák's multiple comparison test. ****$p < 0.0001$.

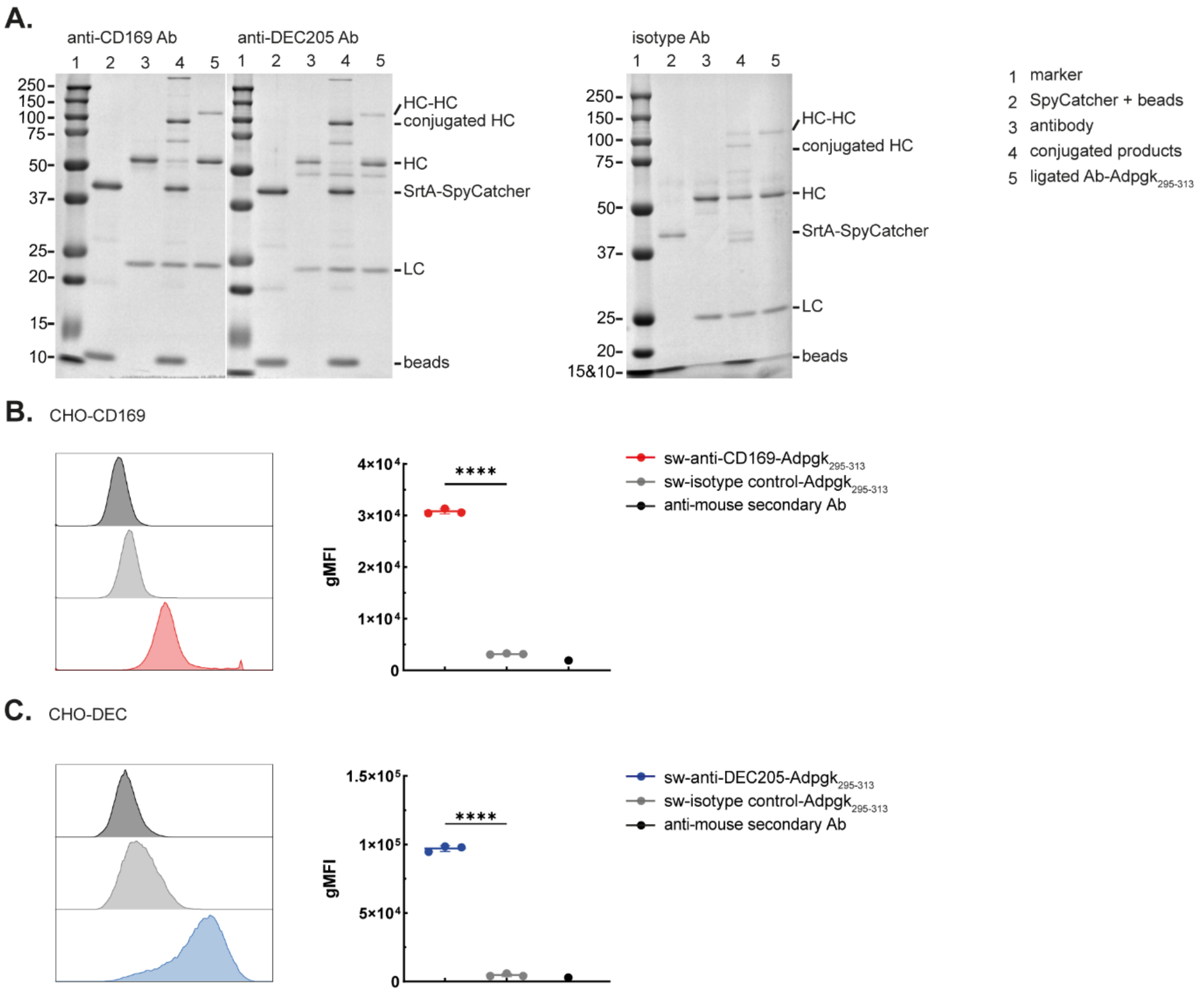

Figure S5. PBSL of Adpgk$_{295\text{-}313}$ peptides to sw- anti-CD169, anti-DEC205, and isotype control mouse IgG2a Abs, Related to Figure 5C. A. SDS-PAGE visualizing the different steps of the PBSL procedure of GGGGG-Adpgk$_{295\text{-}313}$ peptide to the sw-anti-CD169, anti-DEC205, and isotype mouse IgG2a Abs. The cross-linked HC-HC, conjugated HC, original HC, peptide-ligated HC, SrtA-SpyCatcher, and LC were labelled adjacent to the corresponding protein bands on the gel image. C. and D. CHO cells expressing CD169 or DEC205 were stained with PBSL-ligated anti-CD169-Adpgk$_{295\text{-}313}$, anti-DEC205-Adpgk$_{295\text{-}313}$, and isotype-Adpgk$_{295\text{-}313}$ Abs. The gMFI signals of the anti-mouse secondary Ab were determined by flow cytometry. Representative histogram and graphs with mean ± SD from triplicates were shown. Statistical analysis was performed using an unpaired t-test. ****$p < 0.0001$.

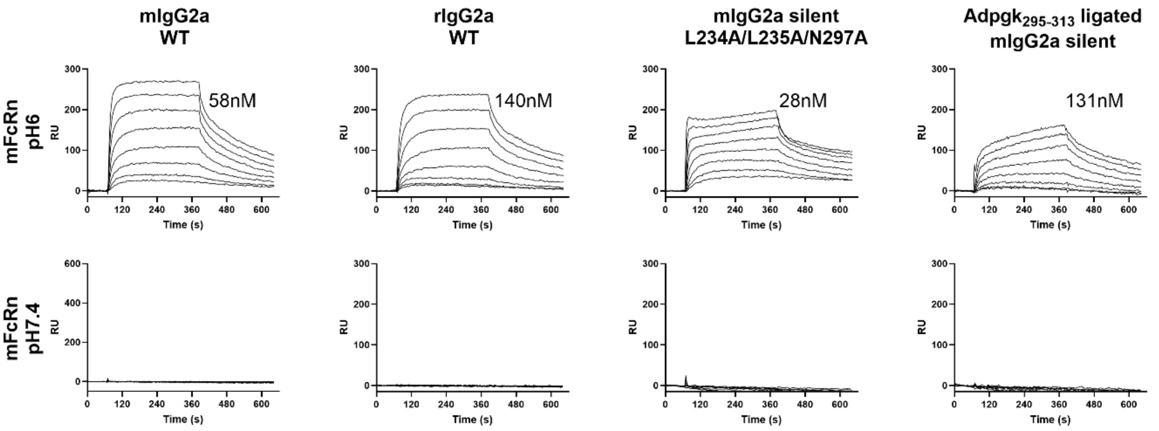

Figure S6. Abs binding affinity to mouse FcRn. Representative sensograms show interaction of mouse (m) IgG2a wild type (WT), rat (r) IgG2a WT, $mIgG2a_{silent}$, and $Adpgk_{295-313}$ ligated $mIgG2a_{silent}$ Abs with immobilized mFcRn at increasing concentrations. Binding to mFcRn is measured in resonance units (RU). Surface plasmon resonance (SPR) was conducted using eight different concentrations of immobilized mFcRn (3.9 nM to 500 nM, following a 2-fold dilution series) to determine affinity K_d of the Fc variants for mFcRn. The mean K_d values were 58 nM for WT mIgG2a, 140 nM for WT rIgG2a, 28nM for silent mIgG2a and 131 nM for $Adpgk_{295-313}$-ligated silent mIgG2a Ab.

Materials and methods

Cloning of donor constructs and SrtA–SpyCatcher

The plasmid pHybr_r2a>m2a(silent)-srt-his (addgene #124807) was digested with BsiWI and SalI to remove the HIS-tag and to replace it with a gene block (IDT) encoding a SpyTag via In-Fusion cloning generating the HDR template plasmid pHybr_r2a>m2a(silent)-srt-SpyT. The pET28a plasmid was digested with XbaI and XhoI to insert a it with a gene block (IDT) encoding a SortaseA-SpyCather003 and Twin-Strep-tag® generating the plasmid pET28a+SrtA-SpyCatcher003-StrepII(2x).

CRISPR/HDR engineering of rat IgG2a hybridomas to produce mouse IgG2a isotype

The engineered hybridomas were produced following the method outlined by van der Schoot et al.[1] Briefly, the HDR template and CRISPR-Cas9 vector (PX458-gR2A_ISO, Addgene #124808) were introduced into cells using the SF Cell Line 4D-Nucleofector X Kit L (V4XC-2024, Lonza). Prior to nucleofection, the cells were washed with phosphate-buffered saline (PBS) containing 0.5% (w/v) BSA. Subsequently, one million cells were resuspended in SF medium, combined with 1 µg of HDR template and 1 µg of the CRISPR-Cas9 plasmid, and transferred into cuvettes for nucleofection using the 4D-Nucleofection System from Lonza (CQ-104, Program SF). After nucleofection, the cells were placed in a six-well plate containing 4 ml of complete medium. The next day, they were transferred to a 10-cm petri dish with 10 ml of complete medium, supplemented with blasticidin (10 µg/ml; 15205-25, Sigma). Antibiotic pressure was sustained for 14 days. Subsequently, antibiotic-resistant cells were clonally expanded by seeding the hybridomas at 0.3 cells per well in F-bottom 96-well plates in 100 µl of complete medium. After an additional 10 days, 3 µl supernatant from wells with a high cell density were spotted on a nitrocellulose membrane and air dried for 30 minutes. The membrane was blocked with PBS/0.1% Tween 20/4% BSA for 45 minutes at room temperature. Subsequently the membrane was incubated with SpyCatcher-mScarlet (30µg/ml, gift from Joen Luirink) in PBS/0.1% Tween 20/0.5% BSA at room temperature. The membrane was washed 3 times for 10 minutes with PBS/0.1% Tween 20 and imaged with the Amersham™ Imager 600. Supernatant from positive clones were individually tested by flow cytometry for reactivity towards their epitope.

Hybridomas culturing, Ab production and purification

Engineered hybridomas specific for DEC205 (NLDC-145),[2] CD169 (clone 3D6) (gift from Prof. Paul R. Crocker, Dundee, Scotland), and isotype control R7D4 were expanded in serum-free medium for Ab

production. Monoclonal Abs were purified from the hybridoma supernatants using Protein G affinity chromatography (GE Healthcare).

Ab binding to CHO cells

CD169- or DEC205-expressing Chinese hamster ovary cells (CHO cells) (gift from Prof. Paul R. Crocker, Dundee, Scotland) were maintained in RPMI-1640 or DMEM (Gibco, Life Technologies) supplemented with 10% FCS (Biowest), 50 U/ml penicillin, 50 µg/ml streptomycin, 2mM L-glutamine (all Lonza) and 1.5 mg/ml G418 (Santa Cruz). The cells were seeded in 96-well plates at a density of 1×10^5 cells per well and incubated for 30 minutes at 4°C with 10 µg/mL of FITC-ligated anti-CD169, anti-DEC205, or an isotype control. For original and sw-Abs, as well as OVA- and Adpgk-ligated Abs, secondary antibodies were used: anti-rat (A11006, Thermo Fisher Scientific) for the original Ab, or anti-mouse (A11017, Thermo Fisher Scientific) for sw-Abs and the ligated Abs. Following two washing steps with PBA (1X PBS, 0.5% BSA, 0.1% sodium azide), cells were fixed with 2% PFA for 20 min at 4°C and measured using an Cytek Northern Lights 3-laser (V-B-R) spectral flow cytometer (Cytek Biosciences, Inc.) and flow cytometry data were analyzed using FlowJo™ v10.10.0 Software (BD Life Sciences).

Peptide Synthesis

The peptides GGGGGK-(FITC), GGGGG-$OVA_{247-279}$, GGGGG-$Adpgk_{295-313}$ were synthesized at the GlycO2peptide unit at our lab by solid phase peptide synthesis using Fmoc chemistry on Liberty Blue microwave assisted peptide synthesizer (CEM Corporation, Matthews, NC, USA). The peptides were purified on a preparative Ultimate 3000 HPLC system (Thermo Fisher Scientific, Breda, The Netherlands) over a Vydac 218MS1022 C18 25 × 250mm column (Grace Davidson, Worms, Germany). Quality control was performed by UPLC-MS on an Ultimate 3000 UHPLC system (Thermo Fisher Scientific) hyphenated with an LCQ-Deca XP Iontrap ESI mass spectrometer (Thermo Finnigan, Waltham, MA, USA) using a RSLC 120 C18 Acclaim 2.2 µm particle 2.1 × 250mm column and ionizing the sample in positive mode.

SDS-PAGE and Western Blot Analysis

Equal amounts (250 ng) of original and isotype-sw-Abs were loaded on SDS-PAGE gels under reducing conditions and visualized by InstantBlue (ab119211, Abcam) or transferred onto polyvinylidene difluoride (PVDF) membranes (Millipore) using a standard transfer protocol. For western blot, the membranes were blocked with 5% non-fat dry milk in 1× TNE-T buffer to prevent non-specific Ab

binding. Abs were then detected using species-specific secondary Abs: Goat anti-Rat IgG (H+L) Cross-Adsorbed Secondary Ab-AF555 (A21434, Thermo Fisher Scientific) for rat primary Abs and Goat anti-Mouse IgG2a Cross-Adsorbed Secondary Ab-AF488 (A21131, Thermo Fisher Scientific) for isotype sw-mouse primary Abs. Abs were visualized using a Sapphire imaging system (Azure Biosystems) to detect the fluorescent signals from the secondary Abs.

Expression of SrtA-SpyCatcher003-(Twin-Strep-tag®) Protein

LOBSTR (BL21DE3) *E. coli* were transformed with the pET28a+SrtA-SpyCatcher003-StrepII(2x) expression vector. Transformed bacteria were cultured in LB medium with 50 µg/mL kanamycin at 37°C until OD600 reached 0.6. The culture was inoculated into auto-induction medium (AIMTB0210, FORMEDIUM) at a 1:500 (v/v) ratio and incubated for 56 h at 24°C with shaking at 220 rpm. Bacteria were harvested, resuspended in lysis buffer (1× PBS, 1% (w/v) OTG, 0.2 mg/mL lysozyme, 20 µg/mL DNAseI, and 1x Roche cOmplete protease inhibitor cocktail), and incubated at RT for 30 min on a head-over-head rotor at 30 rpm, followed by a freeze-thaw cycle. Debris were pelleted at 4500 × g for 30 minutes, and the protein-containing supernatant was stored at -80°C.

PBSL

20nmol SrtA-SpyCatcher003-(Twin-Strep-tag®) protein was captured using 232ul Strep-Tactin® Sepharose® resin (2-1201-010, iba) resulting in a binding capacity of 87.6 nmol/ml bed volume. 10uM of Abs-LPETG-SpyTag and SrtA-SpyCatcher003-(Twin-Strep-tag®) bound beads was incubated to initiate the conjugation step, along with 10 mM EDTA to inhibit SrtA activity. Non-captured proteins and EDTA were removed by washing the beads five times with a buffer volume equal to 10 times the beads' bed volume. To induce peptide ligation to the Abs, 200 µM GGGGG-peptide (20 equiv to Abs-LPETG-SpyTag) and 10 mM $Ca2^{+}$ in 6x beads volume TBS buffer were added to the conjugated Abs. Ligated Abs were released from the resin into the supernatant by centrifugation. The non-ligated free peptides were removed through dialysis against phosphate-buffered saline (PBS) at 4 °C, using a membrane (Spectra-Por® Float-A-Lyzer® G2, Z727040, SigmaAldrich) with a molecular weight cut-off of 100 kDa.

NHS labelling of Abs

Abs were dialyzed against NHS buffer and then mixed with NHS-FITC (Thermo Fisher, 46410) 4mg/ml solution (dissolved in DMSO) at a molar ratio of 1:4 (Ab: FITC) and incubated for 3 h at RT with gentle stirring. The labelled Abs were separated from the free FITC by size exclusion chromatography using a Sephadex G-25 column (GE Healthcare) equilibrated with PBS (pH 7.4)

Affinity measurements of Abs to mouse FcRn

For FcRn affinity measurements, the antibodies were spotted at 5 concentrations in a 2-fold dilution series in duplo starting at 60 nM on a SensEye G Easy2Spot in 10 mM sodium acetate at pH 4.5 supplemented with 0.075% (v/v) Tween-80. Mouse FcRn (Acro biosystems) was injected at 8 concentrations starting from 3.9 nM to 500 nM (2-fold dilution series) in PBS containing 0.075% (v/v) Tween-80. Independent experiments were performed to determine affinities at pH6.0 and 7.4. The sensor surface was regenerated between the cycles by 10 mM Gly-HCl, pH 2.4.

Affinity calculations were carried out by performing an equilibrium analysis interpolating to an Rmax of 200 RU for the FcRn's. A 1:1 Langmuir binding model was used for fitting, assuming equilibrium to be reached after 360s of mouse FcRn containing analyte injections, as described previously.[3] Analysis and calculations were performed using Scrubber Software Version 2 (BioLogic Software).

Animals

C57Bl6/J WT female mice aged 8-12 weeks were purchased from Charles River or bred in-house at the animal facility of Amsterdam UMC, ARIA (location AMC). All animal experiments were carried out in accordance with Dutch government guidelines approved by Animal Experiment Committee (DEC) and Central Committee on Animal Experiments (CCD, AVD11400202216545).

Splenocytes isolation and evaluation of Ab uptake

The spleens were digested using a mixture of 4 mg/ml Lidocaine, 2 WU/ml Liberase TL (Roche, Germany) and 50 µg/ml DNase I (Roche, Germany) for 12 min at 37 °C with continuous stirring. After adding ice cold RPMI-1640 (Gibco, Life Technologies) supplemented with 10% heat-inactivated FCS (Biowest), 10 mmol EDTA, 20 mmol HEPES and 50µM 2-mercaptoethanol. Red blood cells were lysed using ammonium-chloride-potassium (ACK) lysis buffer and splenocytes were filtered through a 70-100µm cell strainer. For *in vivo* Ab uptake, mice were i.v. injected with 20 µg ligated anti-CD169-FITC,

anti-DEC205-FITC, isotype control or NHS-labelled original Abs and the fluorescent signal in multiple splenic immune cell subsets was evaluated 30 min after injection.

Mice Immunization and evaluation of antigen-specific T cells responses

Mice were immunized i.v. in the tail vein with 3 µg of $OVA_{247-279}$ or $Adpgk_{295-313}$ ligated Abs in the presence of 25 µg anti-CD40 Ab (clone 1C10, produced in house) and 25 µg Poly(I:C) (low molecular weight (LMW), InvivoGen, USA). Splenocytes were isolated on day 7 post immunization and stained with $H\text{-}2K^{b}$-$OVA_{257-264}$ tetramer (LUMC, Leiden) or $I\text{-}A^{b}$-$OVA_{262-276}$ tetramer (NIH Tetramer Facility, Emory University, USA). Alternatively, intracellular IFNγ staining was performed following re-challenge with with MHC class I restricted $OVA_{257-264}$ peptide (0.1 µg/ml), or MHC class I restricted $Adpgk_{299-307}$ (5µg/ml for 5 hrs in presence of GolgiPlug (BD Biosciences). Add overnight incubation MHC class II restricted $OVA_{262-276}$ peptide (100 µg/ml) for 24hr with last 5 hr also in presence of GolgiPlug (BD Biosciences).

Statistical analysis

Statistical analysis was performed using one-way or two-way ANOVA test with Šidák's multiple comparison test in GraphPad Prism v10.2.0. Differences were considered to be statistically significant when *p < 0.05, **p < 0.01, ***p < 0.001, ****p < 0.0001. All values are expressed as ±SD and individual mice are indicated.

Sequence of LPETG-SpyTag

ACCTGGTAAACGTACGCTGCCTGAAACAGGCGGAGGAAGCGGAGGTTCTGGCGGTAGTGGTGGATCTGGCG
GATCTGCCCACATCGTGATGGTGGACGCCTACAAGCCCACCAAATGAGTCGACGTCGAGGCCC

Sequence of pET28a+SrtA-SpyCatcher003-StrepII(2x)

ATGCAAGCTAAACCTCAAATTCCGAAAGATAAATCAAAAGTGGCAGGCTATATTGAAATTCCAGATGCTGATA
TTAAAGAACCAGTATATCCAGGACCAGCAACACCTGAACAATTAAATAGAGGTGTAAGCTTTGCAGAAGAAAA
TGAATCACTAGATGATCAAAATATTTCAATTGCAGGACACACTTTCATTGACCGTCCGAACTATCAATTTACAAA
TCTTAAAGCAGCCAAAAAAGGTAGTATGGTGTACTTTAAAGTTGGTAATGAAACACGTAAGTATAAAATGACA
AGTATAAGAGATGTTAAGCCAACAGATGTAGAAGTACTAGATGAACAAAAAGGTAAAGATAAACAATTAACA
TTAATTACTTGTGATGATTACAATGAAAAGACAGGCGTTTGGGAAAAACGTAAAATCTTTGTAGCTACAGAAG
TCAAAGGCGGTTCTGGTGGCAGTGGCGGTAGCGGTGGTAGTGGTGGCAGTGGTTCTGGAATGGTAACCACCT
TATCAGGTTTATCAGGTGAGCAAGGTCCGTCCGGTGATATGACAACTGAAGAAGATAGTGCTACCCATATTAA
ATTCTCAAAACGTGATGAGGACGGCCGTGAGTTAGCTGGTGCAACTATGGAGTTGCGTGATTCATCTGGTAAA
ACTATTAGTACATGGATTTCAGATGGACATGTGAAGGATTTCTACCTGTATCCAGGAAAATATACATTTGTCGA

AACCGCAGCACCAGACGGTTATGAGGTAGCAACTCCAATTGAATTTACAGTTAATGAGGACGGTCAGGTTACTGTAGATGGTGAAGCAACTGAAGGTGACGCTCATACTGGCTGGAGTCATCCTCAATTCGAGAAAGGTGGAGGTTCTGGCGGTGGATCGGGAGGTTCAGCGTGGAGCCACCCGCAGTTCGAAAAATAA

Table S1. Amino acid sequence of substrates

Name	Amino acid sequences	Molecular weight (Da)	Relative MW shift compared to sw-Ab (Da)
**Cleaved off part of Abs**	(LPET)*GGGSGGSGGSGGSGGSAHIVMVDAYKPTK	2536	n.a
**G(5)-FITC**	GGGGGK-(FITC)	790.24	-1745.76
**G(5)-OVA$_{247-279}$**	GGGGGDEVSGLEQLESIINFEKLTEWTSSNVMEERKIK	4167	1631
OVA$_{257-264}$	SIINFEKL	963.13	n.a
OVA$_{262-276}$	EKLTEWTSSNVMEER	1839	n.a
**G(5)-Adpgk$_{295-313}$**	GGGGGHLELASMTNMELMSSIVHQ	2455.8	-80.2
Adpgk$_{299-307}$	ASMTNMELM	1027.2	n.a

*not counted in the molecular weight,

n.a=not applicable

Table S2. Anti-mouse antibodies for staining.

**Antigen/ reagent**	**Fluorochrome**	**Clone**	**Company**	**Panel**
XCR1	BV421	ZET	Biolegend	Uptake
I-A/I-E	BV510	M5/114.15.2	Biolegend	
CD11c	BV650	HL3	BD Biosciences	
F4/80	PE-CF594	T45–2342	BD Biosciences	
Sirp1a	AF700	P84	Biolegend	
TIM4	PE	RMT4-54	Biolegend	
CD8	APC	53-6.7	Biolegend	T cell tetramer staining
H-2K^b/OVA$_{257-264}$	PE tetramer	N/A	LUMC*, Leiden, The Netherlands	
CD3	BV711		Biolegend	
CD11a	FITC	M17/4	eBioscience	
CD4	PE	GK1.5	eBioscience	
I-Ab/OVA$_{262-276}$	APC tetramer	N/A	Tetramer core facility NIH, Atlanta, GA, USA	
CD8	PE-Cy7	53-6.7	BD Biosciences	

IFNγ	APC	XMG1.2	eBioscience	Re-stim intracellular IFNγ staining
CD11a	FITC	M17/4	eBioscience	

* Leids universitair medisch centrum

References

(1) van der Schoot, J. M. S.; Fennemann, F. L.; Valente, M.; Dolen, Y.; Hagemans, I. M.; Becker, A. M. D.; Le Gall, C. M.; van Dalen, D.; Cevirgel, A.; van Bruggen, J. A. C.; et al. Functional diversification of hybridoma-produced antibodies by CRISPR/HDR genomic engineering. *Sci Adv* **2019**, *5* (8), eaaw1822. DOI: 10.1126/sciadv.aaw1822

(2) Kraal, G.; Breel, M.; Janse, M.; Bruin, G. Langerhans' cells, veiled cells, and interdigitating cells in the mouse recognized by a monoclonal antibody. *J Exp Med* **1986**, *163* (4), 981-997. DOI: 10.1084/jem.163.4.981

(3) Dekkers, G.; Bentlage, A. E. H.; Stegmann, T. C.; Howie, H. L.; Lissenberg-Thunnissen, S.; Zimring, J.; Rispens, T.; Vidarsson, G. Affinity of human IgG subclasses to mouse Fc gamma receptors. *MAbs* **2017**, *9* (5), 767-773. DOI: 10.1080/19420862.2017.1323159
